# Double Inferior Vena Cava Diagnosed Following Urinalysis Screening in a 15-Year-Old Boy

**DOI:** 10.7759/cureus.79397

**Published:** 2025-02-21

**Authors:** Shintaro Sugiyama, Shoichiro Kanda, Keiichi Takizawa, Yuko Kajiho, Yutaka Harita

**Affiliations:** 1 Pediatrics, The University of Tokyo, Tokyo, JPN

**Keywords:** asymptomatic hematuria, double inferior vena cava, microscopic hematuria, three-dimensional contrast-enhanced computed tomography, venous anomaly

## Abstract

Microscopic hematuria is one of the most common findings in urinalysis screening. While some cases may be clinically significant, such as early-stage glomerulonephritis, the majority are of limited clinical significance, including asymptomatic hematuria. We report a case where microscopic hematuria was first detected at age 3 and became more pronounced at age 15, leading to further evaluation. Imaging studies revealed a rare congenital vascular anomaly, double inferior vena cava, with narrowing at the junction of the left renal vein and the inferior vena cava. This narrowing caused mild elevation in renal vein pressure, likely contributing to the observed microscopic hematuria.

Repeated positive findings in urinalysis screening provided the opportunity for further investigation, leading to diagnosis. This highlights the importance of considering vascular anomalies in cases of unexplained microscopic hematuria. Comprehensive evaluation, including imaging, can be essential in identifying the underlying cause and guiding management in such patients.

## Introduction

Urinalysis screening is a non-invasive test conducted in various settings, including workplaces and schools. While specific screening criteria may vary across regions, its primary aim is to detect hematuria and proteinuria, facilitating the early identification of conditions that may lead to renal failure, such as congenital anomalies of the kidney and urinary tract, nephritis, and nephrotic syndrome. Some reports suggest that early detection of nephritis through such programs may contribute to reducing cases of renal failure [1,2]. The frequency of serious urologic disease in patients with asymptomatic microhematuria was 2.3% [3]. These cases are typically of minimal clinical significance and are most often managed through careful observation. Indeed, the identification of clinically significant abnormalities through such screening programs remains relatively rare.

Here, we report a case of a patient with recurrent asymptomatic hematuria detected through urinalysis at the age of three. At the age of 15, an increased hematuria was identified, prompting further evaluation, including abdominal ultrasonography, which revealed a rare venous anomaly. We discuss the clinical course and imaging findings and consider the implications for urinalysis screening systems in diagnosing such cases.

## Case presentation

A three-year-old boy was referred to our hospital after urinary screening detected urine occult blood. The patient had no medical history, prior infections, or family history of kidney disease or hearing loss, except for an aunt diagnosed with isolated hematuria.

At the initial visit, the patient’s height was 99.2 cm (-0.4 SD), and weight was 15.0 kg (-0.4 SD), with no evidence of short stature or growth retardation. Blood pressure was within the normal range at 94/78 mmHg. Urine tests at our hospital showed no occult blood, and urine sediment analysis revealed 1-4 red blood cells (RBCs)/high-power field (HPF). Abdominal ultrasonography showed no abnormalities in the kidneys or bladder. The patient was diagnosed with asymptomatic hematuria and followed up periodically.

Subsequently, the patient did not consistently exhibit a positive urinary occult blood reaction during school-based urine screening. The patient visited our hospital approximately once every 2-3 years when a positive reaction was detected. The progress of urinalysis results up to age 14 is shown in Table 1.

**Table 1:** The progress of urinalysis results

Age (years old)	4	11	13	14	15	Reference range
Specific gravity	1.026	1.007	1.013	1.016	1.032	1.005～1.030
pH	6.0	7.0	6.0	5.0	5.5	5.0～7.5
Occult blood	(-)	(+/-)	(+/-)	(2+)	(3+)	(-)
Protein	(-)	(-)	(-)	(-)	(+/-)	(-)
Glucose	(-)	(-)	(-)	(-)	(-)	(-)
Red blood cells (/high power field)	1-4	<1	<1	1-4	5-9	<1
White blood cells (/high power field)	<1	<1	<1	<1	<1	<1
TP/Cre (mg/gCre)	48.08	192.3	78.1	39.0	41.2	<150

Urinalysis showed an intermittent occult blood reaction ranging from +/- to 2+, with less than 4 RBCs/ HPF in the urinary sediment, and no hematuria was detected at our hospital. Therefore, no additional investigations were performed. However, at the age of 15, urinalysis showed an occult blood reaction of 3+ and 5-9 RBCs/ HPF, showing a stronger reaction than previously observed. Considering this exacerbation and the long-term history of urinary abnormalities, further evaluation was conducted, including imaging studies.

**Figure 1:**
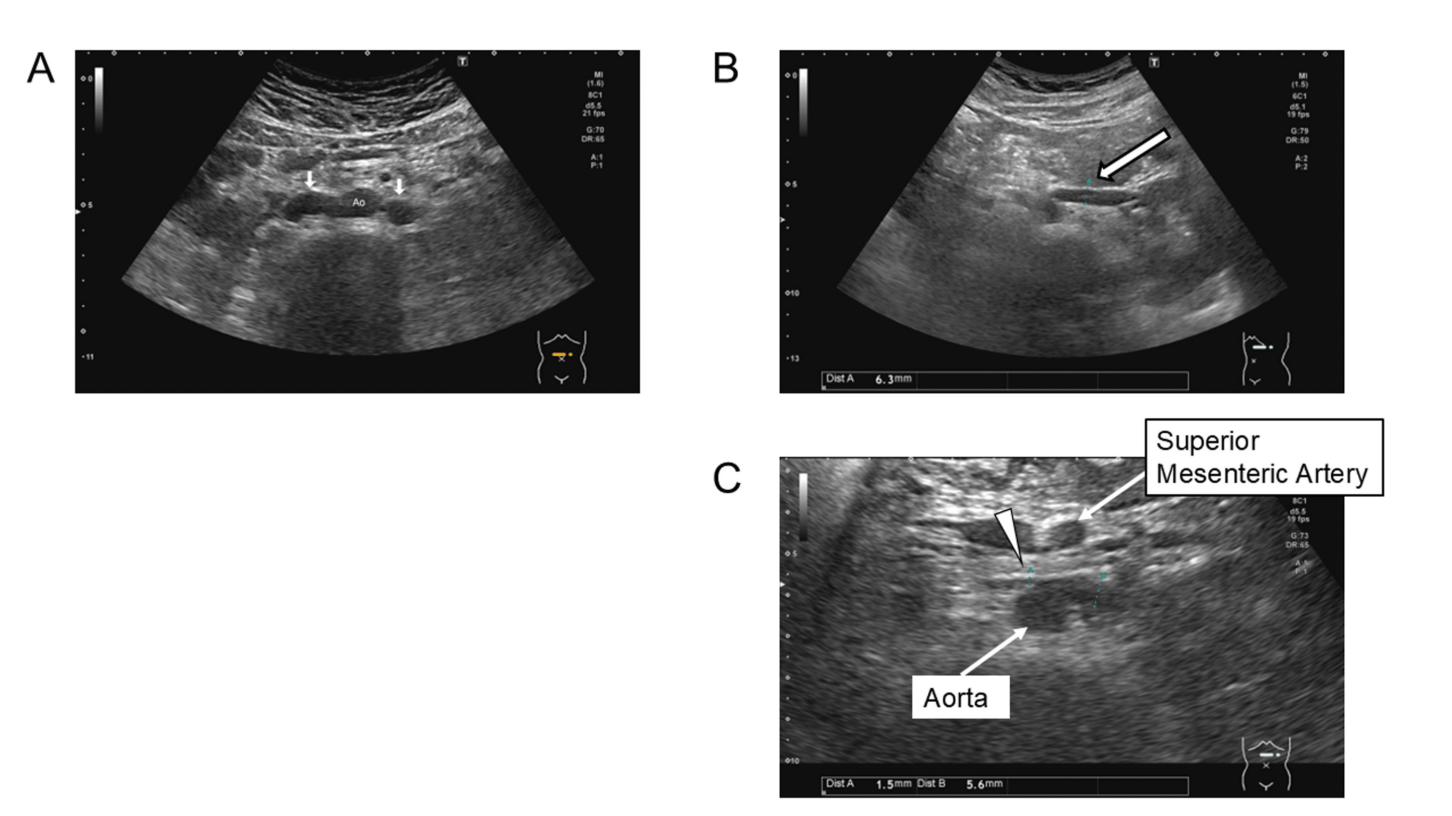
Ultrasound image demonstrating double inferior vena cava. (A) The inferior vena cava (arrows) is observed on both sides of the abdominal aorta. (B) The arrow indicates the diameter of the left renal vein at the renal hilum, measuring 6.3 mm. (C) The arrowhead denotes the diameter of the left renal vein as it courses between the superior mesenteric artery and the aorta, measuring 1.5 mm.

At age 15, the patient’s height was 165 cm (-0.5 SD), weight was 72 kg (+1.2 SD), with an obesity index of +25.2%. Blood tests showed mildly elevated alanine aminotransferase (ALT) levels (51 U/L; reference range: 9-35 U/L) but normal serum creatinine (0.82 mg/dL; reference range: 0.47-0.93 mg/dL). Abdominal ultrasonography revealed fatty liver changes but no significant abnormalities in the kidneys. However, double inferior vena cava (IVC) was detected (Figure 1A).

**Figure 2:**
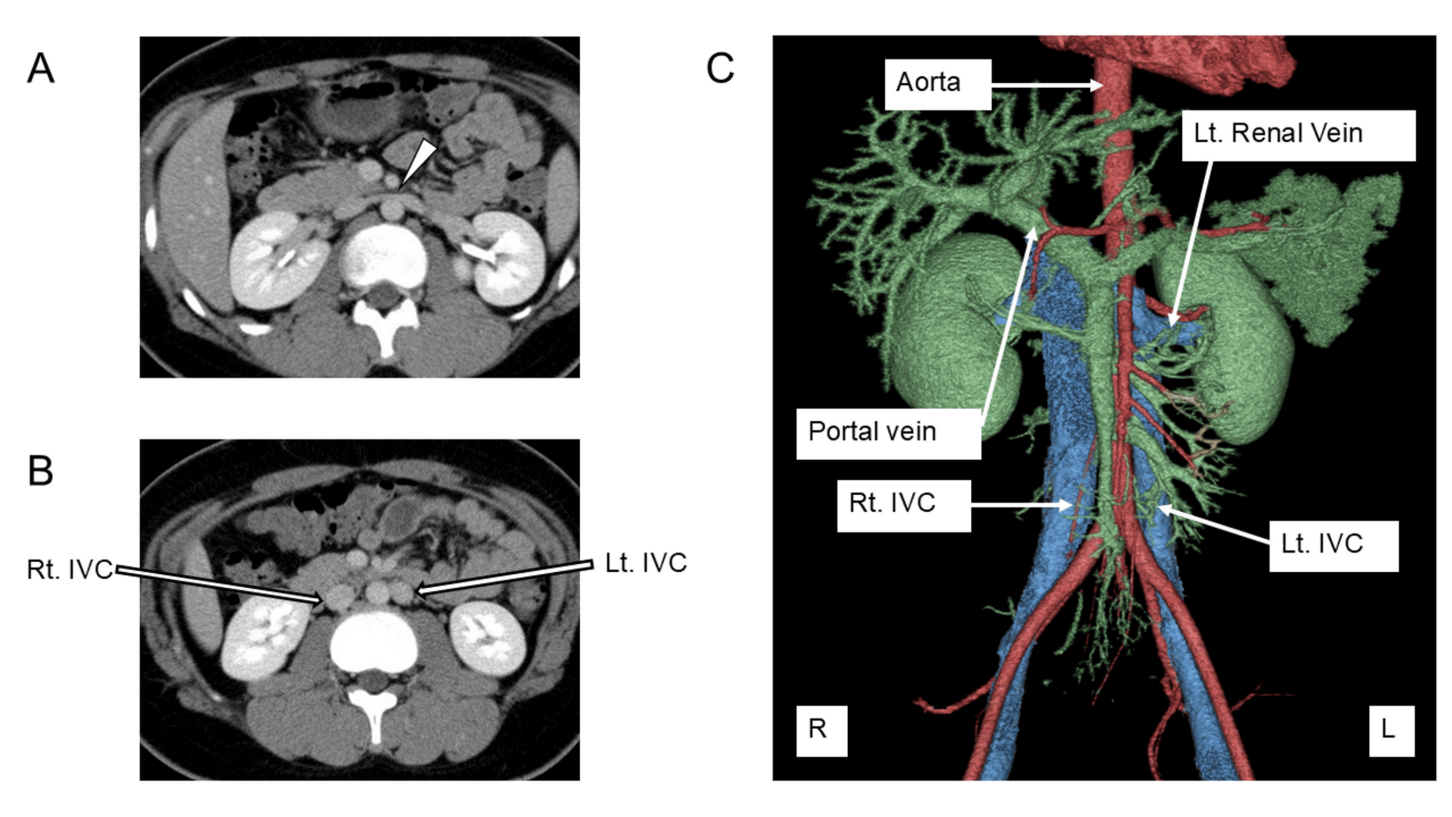
Axial (A, B) and three-dimensional (C) reconstructed contrast-enhanced CT images demonstrating double inferior vena cava. The right and left common iliac veins independently drain into their respective IVC. Notably, compression of a vessel (arrowhead) is observed at the confluence of the left IVC and the left renal vein, anterior to the abdominal aorta. IVC: Inferior vena cava

The diameter of the left renal vein measured 6.3 mm at the renal hilum (Figure 1B) and narrowed to 1.5 mm anterior to the abdominal aorta (Figure 1C), suggesting a pressure gradient of 4.6 mmHg based on a simplified Bernoulli equation. Contrast-enhanced computed tomography (CT) confirmed that the left renal vein joined the left IVC anterior to the abdominal aorta, where the narrowing occurred. The left IVC subsequently joined the right IVC. The left common iliac vein drained into the left IVC and the right common iliac vein drained into the right IVC (Figure 2). No findings suggestive of left renal vein entrapment syndrome, such as reversed contrast flow, were observed. Additionally, no hydronephrosis or urinary tract anomalies were identified.

Based on these findings, elevated pressure in the left renal vein due to compression at the junction of the left renal vein and the left IVC was considered the cause of asymptomatic hematuria. At age 16, follow-up urinalysis revealed no hematuria. As there were no other accompanying symptoms, outpatient follow-up was continued.

## Discussion

This case involves a pediatric patient with recurrent microscopic hematuria detected through urinalysis screening. The patient exhibited no accompanying symptoms, and no hematuria was confirmed at our hospital. Consequently, the patient was diagnosed with asymptomatic hematuria and followed up for observation. At the age of 15, hematuria was confirmed for the first time at our hospital. This prompted further imaging studies, including abdominal ultrasonography, which revealed double IVC.

Although patients with repeated positive urine test results are typically referred to specialists, ultrasonography is not included as a standard diagnostic procedure. Therefore, it is plausible that some patients preliminarily diagnosed with asymptomatic hematuria may have underlying vascular abnormalities, such as in this case.

Double IVC is a congenital vascular anomaly resulting from abnormal development of the IVC system. It occurs in approximately 0.2-3% of the general population [4]. The development of the IVC system occurs between the four and seven gestational weeks, involving the regression and persistence of the posterior cardinal, supracardinal, and subcardinal veins [5]. Typically, the posterior cardinal veins regress, while the subcardinal veins contribute to the formation of the suprarenal segment of the IVC, and the supracardinal veins give rise to the infrarenal segment. When the supracardinal veins persist without merging into a single venous trunk, a double IVC anomaly occurs, most often resulting in the duplication of the infrarenal IVC [6]. Our case represents this typical form of double IVC, where the left IVC terminated at the level of the left renal vein.

Clinically, most cases of double IVC are asymptomatic and are often incidentally discovered during evaluations for other diseases. However, associations with deep vein thrombosis in young patients [7] and increased risk of vascular injury during retroperitoneal surgeries, kidney transplantation, and interventional radiological procedures have been noted [8,9]. Additionally, as seen in this case, hematuria may present as an initial symptom, potentially leading to non-glomerular hematuria. Some cases present with gross hematuria.

Reported pediatric cases of double IVC are limited compared to adult cases, with most diagnoses made incidentally during evaluations for other conditions or after episodes of gross hematuria. In adults, there is a report of microscopic hematuria detected during a health checkup leading to further imaging studies and the subsequent diagnosis of diverticulum-like remnants of the left IVC.

Based on case reports and animal studies, the mechanism by which hematuria occurs in patients with double IVC is suggested to involve increased renal venous pressure due to venous anomalies. Elevated left renal vein pressure has been described in several reports, with one proposed mechanism being the compression of the junction between the left IVC and the left renal vein by the abdominal aorta and the superior mesenteric artery (left renal vein entrapment syndrome) [10]. Alternatively, other studies have suggested that mechanical compression of the junction between the left IVC and the left renal vein as it passes over the abdominal aorta and the spine may be a cause [11]. In this case, contrast-enhanced CT imaging revealed that the distance between the abdominal aorta and the superior mesenteric artery was preserved, and the Nutcracker phenomenon was not observed. However, the junction between the left renal vein and the IVC appeared flattened on the anterior surface of the abdominal aorta, suggesting that the renal venous pressure elevation in this case was more likely due to the latter mechanism.

Experimental studies in dogs have demonstrated that artificially inducing stenosis or occlusion of the renal vein results in hematuria in all cases where renal venous pressure exceeds 30 mmHg [12]. Furthermore, a proportional relationship between renal venous pressure and the number of red blood cells in the urine has been reported. Similarly, in human cases, under normal physiological conditions, the pressure gradient between the IVC and the distal left renal vein typically remains below 1 mmHg. However, in patients with Nutcracker syndrome, this gradient may become markedly elevated, reaching levels of up to 7 mmHg [13]. In the present case, the pressure difference between the renal hilum and the anterior surface of the abdominal aorta was mildly elevated at 4.6 mmHg. Given this mild elevation, hematuria was not constantly observed but rather detected intermittently, approximately once every few years, during urinary screening.

## Conclusions

This case demonstrates the incidental diagnosis of double IVC following the detection of microscopic hematuria through urinalysis screening. The findings suggest that vascular anomalies may be an underrecognized cause of asymptomatic hematuria. Therefore, imaging evaluation, as demonstrated in this case, can be valuable in assessing unexplained microscopic hematuria.
